# Promoting and delivering antenatal care in rural Jimma Zone, Ethiopia: a qualitative analysis of midwives’ perceptions

**DOI:** 10.1186/s12913-019-4596-x

**Published:** 2019-10-21

**Authors:** Nicole Bergen, Alzahra Hudani, Shifera Asfaw, Abebe Mamo, Getachew Kiros, Jaameeta Kurji, Sudhakar Morankar, Lakew Abebe, Manisha A. Kulkarni, Ronald Labonté

**Affiliations:** 1 Faculty of Health Sciences, University of Ottawa, Ottawa, Canada; 2 Department of Health, Behavior & Society, Institute of Health, Jimma University, Jimma, Ethiopia; 3 School of Epidemiology & Public Health, University of Ottawa, Ottawa, Canada

**Keywords:** Health extension workers, Midwives, Teamwork, Antenatal care, Ethiopia

## Abstract

**Background:**

Despite improvements in recent years, Ethiopia faces a high burden of maternal morbidity and mortality. Antenatal care (ANC) may reduce maternal morbidity and mortality through the detection of pregnancy-related complications, and increased health facility-based deliveries. Midwives and community-based Health Extension Workers (HEWs) collaborate to promote and deliver ANC to women in these communities, but little research has been conducted on the professional working relationships between these two health providers. This study aims to generate a better understanding of the strength and quality of professional interaction between these two key actors, which is instrumental in improving healthcare performance, and thereby community health outcomes.

**Methods:**

We conducted eleven in-depth interviews with midwives from three rural districts within Jimma Zone, Ethiopia (Gomma, Kersa, and Seka Chekorsa) as a part of the larger Safe Motherhood Project. Interviews explored midwives’ perceptions of strengths and weaknesses in ANC provision, with a focus as well on their engagement with HEWs. Thematic content analysis using Atlas.ti software was used to analyse the data using an inductive approach.

**Results:**

Midwives interacted with HEWs throughout three key aspects of ANC promotion and delivery: health promotion, community outreach, and provision of ANC services to women at the health centre and health posts. While HEWs had a larger role in promoting ANC services in the community, midwives functioned in a supervisory capacity and provided more clinical aspects of care. Midwives’ ability to work with HEWs was hindered by shortages in human, material and financial resources, as well as infrastructure and training deficits. Nevertheless, midwives felt that closer collaboration with HEWs was worthwhile to enhance service provision. Improved communication channels, more professional training opportunities and better-defined roles and responsibilities were identified as ways to strengthen midwives’ working relationships with HEWs.

**Conclusion:**

Enhancing the collaborative interactions between midwives and HEWs is important to increase the reach and impact of ANC services and improve maternal, newborn and child health outcomes more broadly. Steps to recognize and support this working relationship require multipronged approaches to address imminent training, resource and infrastructure deficits, as well as broader health system strengthening.

## Background

Ethiopia, the second most populous country in Africa, faces a high burden of maternal mortality, with approximately 353 maternal deaths per 100,000 live births in 2015 [1]. While Ethiopia has seen some reduction in maternal mortality over the past decades, [2] its unachieved Millennium Development Goal target of 267 deaths per 100,000 live births remains a stated aim in current national health policies [3]. With the introduction of the Health Extension Program in 2003, [4] Ethiopia prioritized increasing access to essential community-based health services and education in rural areas (where 80% of the population resides [5]), including a focus on improving maternal, newborn and child health (MNCH). Since this time, Ethiopia has seen improvements in several health indicators, including increased utilization of crucial maternal health services [4, 6].

In particular, the expansion of antenatal care (ANC) services into rural areas is a major part of the country’s efforts to tackle preventable maternal mortality and morbidity. ANC, delivered in Ethiopia over four visits (Additional file 1: Table S1), promotes the early detection and treatment of pregnancy-related illness by identifying women who are at a heightened risk of delivery-related complications and ensuring they deliver at an adequately equipped facility [7].^1^ Indeed, in Ethiopia, where home births are common, ANC visits provide an opportunity to emphasize recommendations for all women to deliver in a health facility. The percentage of women who received ANC from a skilled attendant has increased from 27% in 2000 to 62% in 2016, while the percentage of live births delivered at a health facility increased markedly from just 5% in 2000 to 26% in 2016 [8]. Despite these improvements, however, the substantial gap between ANC attendance and facility births is one indication of the need to investigate health promotion initiatives and the delivery of ANC [9].

The quality of the user-provider interaction is a large concern for women in their decisions surrounding ANC. Previous research about ANC services in urban areas of Jimma Zone, Ethiopia, has found the high turnover of health workers to be a barrier, exacerbated by a focus on training students, which hinders the continuity of care and privacy [10]. Interventions aimed at strengthening ANC delivery through targeted technical training, equipment provision and guideline adaption have had only mixed success in strengthening the quality and frequency of ANC visits [11, 12].

The provision of ANC services in rural areas of Ethiopia is the joint responsibility of midwives and Health Extension Workers (HEWs). Midwives, stationed in rural areas at Primary Health Care Units (PHCUs), are frontline health workers who provide basic emergency obstetric and newborn care during normal deliveries, and identify and refer complications of childbirth to higher levels of the health system [13, 14]. Midwives based at PHCUs supervise the work of several HEWs stationed across about 5 health posts (the most decentralized health facility, staffed by HEWs). When trained to international standards, midwives can provide nearly 90% of care needed by women and newborns [15]. In Ethiopia, about 97% of health centres or clinics employ at least one midwife, although few of these midwives have received training in basic emergency obstetric and newborn care [16].

HEWs play a significant role alongside midwives to promote maternal health [4]. These community-based health workers, all female, are recruited from local villages and have at least a grade ten level of education before undergoing their one-year HEW training [4]. Once assigned at to their health post, HEWs are expected to spend 75% of their time conducting community outreach activities [4, 17], and serve a critical role in connecting women from their respective communities to higher levels of the health system. HEWs have a role in identifying, training and collaborating with the voluntary Women Development Army, a group of community leaders who support the work of the HEW. In terms of promoting MNCH, HEWs fulfill tasks such as: regular mapping of households with pregnant women; providing referral linkage for ANC visits at the PHCU; individual counselling on nutrition, breastfeeding, immunization, insecticide-treated net use, and family planning; and emphasizing the importance of institutional delivery through educational conferences [18]. As of 2013, there are about 38,000 HEWs deployed across 16,440 rural kebeles (villages of about 5000 people) in Ethiopia [19, 20].

Midwives and HEWs work together in different aspects of ANC delivery to ensure women receive adequate ANC services. These efforts are represented across three noteworthy stages of ANC service promotion and delivery: health promotion (e.g. leading pregnant women conferences [21]); community outreach (e.g. identification and follow-up of pregnant women); and ANC visits. It is unclear, however, how midwives and HEWs have been progressing in collaborative efforts to provide ANC to women. Collaboration among key actors is critical in improving productivity and performance in the promotion and delivery of ANC services, and thereby maternal and neonatal health outcomes [22, 23]. Evidence from mid and high-income workplace settings suggest that team cohesion is associated with improved organizational performance and learning [24]. Studies also suggest that collaboration among interdisciplinary health care teams contributes to improved health outcomes among populations in healthcare settings [25], and improved performance through the utilization of collective knowledge [26].

This article focuses on such collaboration using data collected from midwives who not only have a large role in ANC service delivery, but also work alongside HEWs to promote the use of MNCH services, including ANC, at the community level. The aim of the study is to generate a better understanding of the interaction between midwives and HEWs in promoting and providing ANC, in order to provide new insights for decision-makers to build on current strengths, address barriers, and consider ways forward in improving maternal healthcare utilization and quality of care. Explorations of midwife perspectives on ANC service provision in rural Ethiopia is currently understudied, though important to discern the way forward for strengthening MNCH in rural settings.

## Methods

### Study context

Evidence for this article is derived from the larger Safe Motherhood Project, a mixed-methods, randomized cluster intervention trial testing the roll out and scale up of interventions to improve access to health facilities for pregnant women and reduce preventable maternal and neonatal morbidity and mortality. The study focuses on two interventions: the delivery of information, education, and communication workshops in conjunction with key community leaders; and improving the functionality of maternal waiting areas (residences nearby health facilities for women nearing their delivery date, allowing for close monitoring and rapid birth attendance by a skilled midwife [25]). This research is being conducted in Jimma Zone, Ethiopia, across 24 PHCUs in three rural districts: Gomma, Kersa, and Seka Chekorsa. There are 112 kebeles in the study area (41 in Gomma, 32 in Kersa and 38 in Seka Chekorsa).

### Data collection

As a part of the larger Safe Motherhood Project baseline qualitative assessment, we conducted semi-structured, in-depth interviews with midwives, HEWs, PHCU directors, male and female community leaders, and religious leaders to explore various aspects of MNCH in the community. The interviews included questions about: MNCH service delivery and use; stakeholder roles in promoting MNCH; community participation in MNCH activities; problems related to MNCH; and maternity waiting areas. This paper focuses on data collected from 11 midwives, employed at PHCUs across Gomma (*n* = 5), Kersa (*n* = 2) and Seka Chekorsa (*n* = 4) districts. Findings derived from the HEW interviews (*n* = 31) have been reported elsewhere [27]. The interview guide used in this study was developed for the Safe Motherhood Project (Additional file 2).

For interviews with midwives, six random PHCUs for each intervention arm were selected using the Wolfram Alpha software. This software generated sets of six random and different integers, which were matched with PHCU listing numbers to randomly select PHCUs in each arm for midwife interviews. In total, 18 interviews were to be conducted with PHCU midwives in accordance with the sampling frame. Data collectors were able to conduct 11 of these interviews, achieving 61% of their target. The interviews took place in selected areas in each respective PHCU where the midwives worked.

The baseline qualitative data were collected over a four-month period (November 2016 – February 2017), working with data collectors from Jimma University. (A parallel baseline quantitative survey was also administered, but is not reported on in this article.) The data collectors were fluent in both English and the local language (Afan Oromo), had graduate-level university training, and had previous experience doing qualitative research. The data collectors participated in a one-week training and induction workshop prior to the field work, which focused on techniques to conduct interviews and keep field notes. At least two researchers from the Safe Motherhood Project were present in a supervisory capacity during the field work to address any issues that arose. A member of the research team obtained informed written or oral consent from all study participants. With the permission of the participants, interviews were digitally recorded. The data collectors transcribed and translated the interviews. Detailed field notes were taken by the data collectors and their supervisors to contextualize and supplement the interview data.

### Data analysis

Following detailed readings of the transcripts, a code guide was developed for analysis of all the qualitative baseline data, and continually refined to allow for emergent themes. Data analysis was undertaken by members of the research team, using Atlas.ti software. After summarizing descriptive findings according to the coding categories, major themes and findings from the midwives’ transcripts were identified and discussed by the research team. This inductive approach to analysis facilitated new insights into the data, such as the highly significant and focal aspect of midwife-HEW interaction and engagement surrounding the provision of ANC services (reported in this article).

We adopt a simple health systems analysis framing construct to present the results of the analysis (context – inputs – process – outputs) [28]. First, highlighting context, we begin with midwives’ descriptions of their and HEWs’ extant roles across the three stages of ANC promotion and delivery: health promotion, community outreach and ANC visits. Then, turning to midwife-HEW interactions, we present midwives’ perceptions of the strengths and weaknesses related to the inputs, processes and outputs of ANC delivery.

Ethical clearance was obtained from the authors’ respective institutes: Jimma University College of Health Sciences Institutional Review Board, and University of Ottawa Health Sciences and Science Research Ethics Board.

## Results

### Context: roles in ANC service promotion and delivery

Although midwives take on a supervisory role when working with the HEWs, the HEWs take a leadership role in their local catchment areas. During the first stage of health promotion, HEWs and midwives work collaboratively to plan educational conferences for pregnant women, and counsel women and their husbands. These conferences are intended to take place twice per month at the kebele level, although midwives acknowledge that they may occur less frequently. According to a midwife from Gomma district,

*“They [HEWs] make the participants ready for us and [we come] there and awareness creation is conducted.”*

Both midwives and HEWs appear to be involved in promoting ANC services, and health facility deliveries. The health promotive role of HEWs appears to be especially prominent early in a women’s pregnancy when they promote the timely use of ANC services. A midwife from Kersa acknowledges that efforts to generate community participation yield higher use of MNCH services:

*“Those [midwives and health workers] who have highly participated in the community [will see] client flow increase from day to day, and the confidence of the community will increase, for example [evidenced by higher use of] ANC, long term family planning, abortion care and child immunization.”*

In the second stage of community outreach, HEWs reportedly have a leadership role in identifying pregnant women in the community (often in conjunction with members of the Women Development Army), and report this list to the midwives on a monthly basis. HEWs are also responsible for following up with the women, and appropriately referring them to a midwife at the health centre for first and fourth ANC visits, or as necessary to address complications.

In the third stage, ANC visits, HEWs and midwives have distinct and complementary responsibilities in delivering care at different points of the pregnancy. Women are advised to attend a total of four ANC appointments over the course of their pregnancy: midwives administer the first and fourth ANC visits, while HEWs provide the second and third (in the case of a pregnancy with no apparent complications). During the provision of ANC services and up to the time of delivery, however, the overlapping roles of the midwives and HEWs becomes more evident, as one midwife from Gomma district explains:

*“After the fourth ANC checkup we [midwives and HEWs] prepare them [pregnant women] for delivery and during delivery there is ambulance service. They call to the Health Extension Workers for ambulance service and if their [the HEW’s] phone is not functioning, they can either call me, or the health center director.”*

### Inputs: resources

Midwives identified resource scarcity as a challenge that negatively impacted their interactions with HEWs. In many cases, midwives spoke about barriers related to a lack of human, material, and financial resources, as well as inadequate training or general capacity. They explained that service demands on midwives could create a communication gap between themselves and the HEWs, as a midwife from Seka Chekorsa district noted:

*“My working linkage with the kebele HEWs is too small because I am very busy here at the health centre and there is a gap between me and them.”*

Most of the midwives also reported that a shortage in material resources hindered their ability to adequately perform their role in ANC service delivery, such as running out of ANC cards at the PHCU, which are used to track the services that the women receive. Relatedly, some midwives mentioned that HEWs did not always track the ANC services that they delivered at the heath post, or that HEWs did not always report these activities to the midwives. This made it difficult for midwives to understand the needs of the pregnant women, and appropriately refer women to other health facilities. A midwife from Seka Chekorsa district explains:

*“HEWs have basic data about the ANC [visit], but there is a problem of reporting by them. The problem is that HEWs are not always available at the health post. … [So] we lack good combination on referral issues … because there is a problem of reporting on a continuous basis.”*

As a way to strengthen the linkages with HEWs, many of the midwives emphasized the need to improve the material and human resources at the health facilities. According to the participants, increased resource availability would also help to improve the quality of care that midwives could provide, ameliorating women’s dissatisfaction with ANC services (reported elsewhere [29]). This, in turn would strengthen HEW’s confidence in referring pregnant women from the health post to the PHCU, and make it more worthwhile for women to travel to the health centre for at least two of their four recommended ANC visits.

Similarly, a few midwives stated that receiving basic emergency obstetric and newborn care training in addition to other related training would be beneficial in supplementing the current referral process, and increasing their confidence in service delivery. A midwife from Gomma district explains:

*“It would be better if training was available regularly. Health issues are dynamic, so it is better if when a new thing about health is identified, it [training] reaches health professionals on time, so the way they address the [health] problem [would be] improved.”*

### Process: engagement and communication

Midwives reported meeting with HEWs on a monthly basis when HEWs visit the PHCU for training programs or to collect supplies for the health post. These visits provide an opportunity for midwives and HEWs to discuss women who are not attending ANC services. A midwife from Gomma district explains:

*“ … when there are dropouts they [HEWs] look for them [the women] and communicate their findings with us.”*

Some midwives reported that they, together with HEWs, develop systems to minimize the number of women dropping out from ANC service delivery. A midwife from Gomma district explains the process that she used:

*“We have list of pregnant women, [prepared] with the help of HEWs... If the woman does not come back to the health center, we identify where she went, with the help of the HEWs. And if the women did not yet give birth, we encourage her to continue ANC visits until delivery … So, we communicate with HEWs to trace those women who have dropped out from the visit.”*

In addition to face-to-face meetings or interactions during ANC promotion at the health post level, midwives described modes of communicating with HEWs, including supervisory visits to the health posts. As one midwife from Gomma district describes:

*“There is a linkage: the HEWs invite midwives [to the health post] every two weeks. We go there having developed* a *checklist and then we identified [any] problems. After we identified the problem by using checklist, we identify the good things they’ve done well and the gaps. We give them feedback.”*

This midwife provides examples of other ways that they communicate with HEWs. In cases where they are unable to personally visit the health post, midwives communicate by phone, or through another PHCU health worker assigned to visit the kebele (since midwives themselves rotate across about five health posts).

Alongside these positive aspects of communication, however, midwives frequently reported a fundamental misunderstanding in the roles and responsibilities regarding activities that overlap with HEWs – reflecting a potential deficit in training and job preparation, or a breakdown of communication. As a result, midwives felt that HEWs did not always assist them in duties that are expected to be collaborative efforts. A midwife from Seka Chekorsa commented that this misunderstanding could be due to the HEW’s lack of experience or capacity. When probed further, the midwife responded:

*“They believed that the [ANC] services is only for midwifery workers [to do], therefore some of them never want to assist us.*”

### Outputs: collaborative efforts to increase ANC demand, use and quality

Many midwives observed that working with HEWs to raise awareness about ANC greatly increased demand for all four recommended ANC visits. A midwife from Gomma district commented:

*“Now since conferences [with pregnant women] are continuously held and awareness is created and they [pregnant women] are given [maternal health] services free of payment … there is a [greater] load on ANC service.”*

Because HEWs work in the community and have frequent contact with the women in this setting, they are more likely to encounter undetected pregnancy-related complications that they can then report to the midwives. This frontline interaction is a key strategy for the early detection and prevention of adverse health consequences. A midwife from Gomma district explains how HEWs can facilitate access to higher levels of the health system using ambulance services:

*“After the ambulance services began, we have standby ambulance at our health center. When they [HEWs] call the ambulance, it can go up to the health centre with the professional who is managing the issue and pick them and bring [the woman] to the health center.”*

Several midwives acknowledged well-known barriers to ANC service use, such as distance and lack of transportation. A midwife from Gomma district recognized that working together with HEWs helps women overcome these barriers:

*“We found a solution for this as [pregnant women] attend the first ANC [at the PHCU then], we allow her to attend the other ANC [visits] with the Health Extension Worker [at the nearby health post].”*

Furthermore, some midwives believe that further collaboration between HEWs can strengthen the quality of care that midwives are able to provide. A midwife from Gomma district says:

*“We have a plan in strengthening the linkage with Health Extension Workers for the future.”*

## Discussion

In this study, we explored midwives’ perceptions of ANC provision, including the interaction between two pivotal actors, namely midwives and HEWs. Our findings underscore the complementary and, at times overlapping, roles of midwives and HEWs across aspects of ANC promotion and delivery. While midwives acknowledged many positive aspects of this working relationship, the results also highlight the problematic impact of resource deficits, poor communication channels, and other situational barriers. Addressing these factors stands to benefit the collaborative potential of midwives and HEWs, and thereby enhance the reach, quality and effectiveness of ANC services.

A significant challenge identified by the midwives is the human resources for health shortage, often causing worker burnout and inadequate performance. Although the health worker density in Ethiopia has increased from 0.84 to 1.3 per 1000 population between 2008 and 2013, the doctor, health officer, nurse and midwife to population ratio is only 0.7 per 1000 population – far behind the recommended 2.3 per 1000 required to ensure high coverage [3]. Although some efforts are in place by the Ethiopian government to increase the quantity of higher level health workers to meet the country’s demand – and indeed the early success in scaling up of HEWs has paved the way for more costly initiatives, such as scaling up midwives [13, 30] – the capacity and readiness of higher education institutions to assure quality education has not developed proportionally to its growth in numbers [3]. Many women in Ethiopia still give birth in the home, attended by non-skilled personnel such as traditional birth attendants, family members or neighbours [9].

Enhancing the effectiveness of supervisory activities offers another possible way to improve worker performance in the short term, while serving as a mechanism for professional development, improving health workers’ job satisfaction, and increasing motivation [31, 32]. When delivered in a manner that reinforces trust and mutual respect, supportive supervision contributes to establishing leadership for improved ANC delivery [10]. The main challenges with supervision, also evident in the results of this paper, include improving the quality of supervision and increasing the time supervisors spend with health workers. To the extent that midwives see themselves, and are seen by others, as offering supervision to HEWs, it is important to assess whether they have the sufficient skills, tools, training, and transportation to provide such supervision, and are not over-burdened with other responsibilities.

Also evident from our findings is the perception of midwives as being responsible for managing ANC activities such as the educational pregnant women conferences. Although HEWs are recognized as leaders in their local kebeles and have a leadership role with regards to the activities of the Women Development Army, midwives perceive themselves to be leaders among the HEWs. This hierarchy could prompt a competitive rather than collaborative relationship when both are working together at the health post level. Previous studies have also raised the hierarchical nature of health workers relationships, and the reluctance of lower-ranked health workers to question authority [33].

The different approaches to recruiting and training midwives and HEWs position them differently with regards to the communities that they serve. While HEWs are recruited from the community and assumed to be de facto representatives, midwives are university trained and may be deployed throughout the country. In providing ANC, our findings confirm that HEWs (and, more locally, members of the Women Development Army) generally have a larger role in community outreach than do midwives, and that they are seen as trusted sources of health information [34]. Though HEWs receive a salary, albeit modest, they are assumed to be working in the interest of their communities, whereas it is less apparent how the community views the motivation behind midwives’ engagement – a factor that may influence community members’ willingness to visit midwives for and comply with their advice and recommendations.

A more comprehensive and focused strategy to address the challenges that midwives face as they work together with HEWs to provide ANC is needed, as the role of both actors in improving service utilization among pregnant women are interdependent. Midwives in this study identified some shortcomings in how HEWs delivered on their role with regards to ANC service provision, though nevertheless saw the importance of strengthening this collaboration as the way forward in improved MNCH. Given that there are no national guidelines for ANC in Ethiopia, [10] the lack of formal clarifications surrounding the roles and responsibilities of midwives and HEWs may contribute to miscommunications between the two groups of professionals. In Ethiopia, midwives generally report low job satisfaction, though positive interpersonal relationships with coworkers is a determinant of higher satisfaction [14, 35]. In the rural setting where this study was conducted, the benefits from enhancing the collaborative potential of midwives and HEWs serves as an important aspect of further inquiry in strengthening ANC services and MNCH more generally.

This study has illustrated the direct and indirect implications of broad health system deficits (e.g. resource scarcity, poor infrastructure and inadequate training) on one aspect of MNCH care provision. To address these cross-cutting factors, engagement from high-level political leaders across health and non-health sectors is warranted. Engaging with political leaders has shown to incur promising outcomes with the scale up of education and training programs for health workers [36]; and should continue going forward. The Global Strategy on Human Resources for Health: Workforce 2030, for example, identified political engagement and financial investment as critical factors in building capacity among health workers in settings such as rural Ethiopia [37]. In line with the 2001 Abuja Declaration call for increasing spending on health, Ethiopia has increased its prioritized spending on health over time, yet remains below the specified targets [38]. Although the Ethiopian government has prioritized health, more attention is needed towards key areas of concern as identified by the World Health Organization, which include: funding inconsistency, budget underspends, and misallocation of resources [39].

### Study strengths and limitations

This research explored the perspectives of midwives using semi-structured in depth interviews, which allowed for data collectors to ask open-ended questions and further probe to receive rich insight on their topics of inquiry. Collaborating with local researchers to collect data helped to lessen some level of social desirability bias, which has been raised as a potential concern in qualitative health research [40]. Strategies to minimize social desirability bias were discussed and implemented by the researchers at various stages of the fieldwork. Some of these included modified questioning techniques, and building rapport with interviewees.

Some potential limitations to this research include those that are challenging to fully control in several types of qualitative research. First, transcription errors such as accidental and/or unavoidable alterations of data may have occurred [41]. Moreover, differences in fluency of the English language led to some interpretative problems during data analysis. These issues were generally mitigated by cross-referencing with data collectors and other researchers who were on the field during data collection to clarify linguistic challenges that had surfaced throughout. We acknowledge that data analysis is subject to individual interpretive processes, which are shaped by their unique experiences and perspectives. Researchers remained reflexive throughout the analysis process, and engaged in discussions with local MNCH experts, data collectors and other members of the research team in interpreting the findings.

## Conclusion

The expansion of health services into rural Ethiopia over the past decades demonstrates a commitment to improving MNCH outcomes. There remains a need for more financial, material, and human resources and their strategic deployment to reduce challenges that health workers face in providing ANC and aspects of MNCH care. Influential stakeholders such as the Ethiopian government, and various non-governmental organizations have a role to play in resolving these barriers and establishing more favourable conditions for midwives and HEWs to enhance their collaborations. This may include, for example, integrated trainings to advance key skills and competencies required in their roles, and strategizing on how to improve their collaborative efforts via more regular forms of communicative exchange. Moving forward, we call attention to the need to expand upon this research to further explore the interaction and collaboration between health workers in rural Ethiopia and better understand how they prioritize tasks and overcome barriers in their work.

## Supplementary information

**Additional file 1: Table S1.** Antenatal care service provision in rural Ethiopia. This table shows the recommended timing of four antenatal care visits, including the provider and the major services provided. (DOCX 14 kb)

**Additional file 2.** Overview of in-depth interview guide with midwives. This file outlines the five question sets that guided the in-depth interviews with midwives, including questions and prompts. (DOCX 18 kb)

## Data Availability

Data analyzed for this article are available from the corresponding author on reasonable request.

## Abbreviations

ANC: Antenatal care
HEW: Health extension worker
MNCH: Maternal, newborn, and child health
PHCU: Primary health care unit

## Footnotes

1 Note that, as of 2016, the World Health Organization recommends a minimum of eight ANC visits to reduce perinatal mortality and improve women’s experience of care. Current Ethiopian MNCH policies and practices emphasize four ANC visits, which is a key indicator used for monitoring and target setting.
