# Supplementary material for: Promoting and delivering antenatal care in rural Jimma Zone, Ethiopia: a qualitative analysis of midwives’ perceptions
Source: BMC Health Serv Res. 2019 Oct 21;19:719. doi: 10.1186/s12913-019-4596-x (PMC6805645; doi:10.1186/s12913-019-4596-x)
Supplement: Supplementary file 1 — Additional file 1: Table S1. Antenatal care service provision in rural Ethiopia. This table shows the recommended timing of four antenatal care visits, including the provider and the major services provided. (DOCX 14 kb) [file 12913_2019_4596_MOESM1_ESM.docx]

Appendix table 1

Appendix table 1. Antenatal care service provision in rural Ethiopia (1)

| ANC visit | Recommended timing | Provider | Major services provided* |
| --- | --- | --- | --- |
| 1 | 16 weeks (4 months) | Midwife | -Confirm pregnancy and estimated date of delivery  -Full physical examination (including vital health indicators such as blood pressure, height and weight)  -Basic laboratory tests (including blood tests and HIV screening)  -Iron folate supplements and nutrition advice  -Administer tetanus toxoid vaccine  -Discuss pregnancy history and assess/screen for danger signs and risk factors (including multiple pregnancies, anemia and hypertension)  -Develop birth and emergency plan  -Refer to higher levels of the health system, as required |
| 2 | 24-28 weeks (6 months) | Midwife or HEW | -Monitor pregnancy progress  -Measure and record vital health indicators  -Ensure receipt of vaccine and continued use of iron folate supplements  -Assess/screen for danger signs and risk factors  -Review and modify birth and emergency plan  -Refer to higher levels of the health system, as required |
| 3 | 30-32 weeks (8 months) | Midwife or HEW |  |
| 4 | 36-40 weeks (9 months) | Midwife |  |

*Note that this is not an exhaustive list of the services that may be provided at ANC visits.

1. Federal Ministry of Health Ethiopia. Second generation Health Extension Program: Maternal and infant health care package. Addis Ababa: Federal Ministry of Health Ethiopia; 2018.
