# Supplementary material for: Promoting and delivering antenatal care in rural Jimma Zone, Ethiopia: a qualitative analysis of midwives’ perceptions
Source: BMC Health Serv Res. 2019 Oct 21;19:719. doi: 10.1186/s12913-019-4596-x (PMC6805645; doi:10.1186/s12913-019-4596-x)
Supplement: Supplementary file 2 — Additional file 2. Overview of in-depth interview guide with midwives. This file outlines the five question sets that guided the in-depth interviews with midwives, including questions and prompts. (DOCX 18 kb) [file 12913_2019_4596_MOESM2_ESM.docx]

Supplementary file 1. Overview of in-depth interview guide with midwives

# Question set 1. MCH services at the PHCU level

1. What is the role of the Primary Health Care Unit (PHCU) in managing and/or delivering maternal and child health services?
2. What types of resources does the PHCU commit to providing maternal and child health services?

*Prompt: financial, personnel, materials, training, other*

1. Considering the types of maternal and child health services and activities that are available at the PHCU, where do you feel you are doing well? Where do you feel you could improve?
2. Are there any families for whom these services are especially important? Please explain.
3. Do you target any groups of women in particular? If so, how? Can you think of any examples?

*Prompt: vulnerable population*

## Question set 2. Community participation in MCH activities and services

1. How do community members become aware of MCH services?

*Prompt: use of marketing, media, word of mouth, common practice, etc.*

1. Are community members encouraged to participate in activities related maternal and child health services, in ways like planning, promoting, financing or providing feedback? If so, please explain how this occurs.
   1. What do you think motivates the women in this health district to participate in MCH activities or services?
   2. Why do you think might women decide to not participate? Do you address these factors? If so, how?
   3. Do you at the PHCU encourage participation in any way? If so, how?
   4. How important is it that they participate? How do you think this participation affects maternal and child health outcomes?
   5. What examples of this participation have you experienced?

Question set 3. MCH services across different stages of childbearing

a) I would like us to address experiences related to antenatal care (ANC).

1. What challenges do PHCU staff face in providing ANC?
2. We know that some women attend ANC visits, while others do not. Some may drop out and not complete the four recommended visits. What do you think are the reasons why women may not attend ANC visits?

*Prompt: Reasons for not attending the first visit? Reasons for dropping out?*

1. What do PHCU staff do to encourage attendance and minimize drop outs?
2. Together with staff at the PHCU, HEWs also have a role in the community in providing and promoting ANC visits. What challenges do you think HEWs face, with regards to providing and promoting ANC visits?
3. How do midwives and HEWs work together to promote full ANC visit coverage?
4. Turning now to services related to child birth, I would like to know more about the experiences of women in the PHCU’s catchment area.
5. To begin, what are the factors for a woman to give birth at a health facility?
6. What are factors why a woman may not give birth at a health facility?
7. What do PHCU staff do to encourage women to give birth at a health facility?

*Prompt: challenges faced?*

1. What do HEWs do to encourage women to give birth at a health facility?

*Prompt: challenges faced?*

1. Now let’s talk about the experiences of women in the kebeles of this PHCU during the month after they give birth, with regards to their health and the health of their baby. Postnatal care (PNC) refers to visits with health workers or other types of health services that the mother or baby receives during this period.
2. What challenges do PHCU staff face in providing PNC?
3. Some mothers and their babies may have PNC visits with health workers, while others may not. What do you think are the reasons why mothers and babies may not use PNC?
4. What are reasons why mothers and babies may use PNC?
5. What do PHCU staff do to encourage mothers and babies to make PNC visits?
6. What is the role of the HEW in terms of encouraging mothers to make PNC visits?
7. Thinking about MCH services related to these stages of childbearing (ANC visits, delivery and PNC services), what more could be done to encourage women to use MCH services?

*Prompt: What would be needed to achieve this?*

1. Are there some kebeles that are doing a better job than others at encouraging the use of MCH services (ie, related to ANC, child birth and PNC)?

*Prompt: Can you give an example of how a kebele is performing well? And example of a kebele that is performing less well?*

Question set 4. Health problems

1. What types of serious health problems does PHCU staff or HEWs see during pregnancy?
2. How is the problem addressed? By whom?
3. What improvements could be made to better address the problem?
4. What would be needed to make these improvements? By whom?
5. What types of serious health problems do PHCU staff or HEWs see during childbirth?
6. How is the problem addressed? By whom?
7. What improvements could be made to better address the problem?
8. What would be needed to make these improvements? By whom?
9. What types of serious health problems do PHCU staff or HEWs see during the two days following childbirth?
10. How is the problem addressed? By whom?
11. What improvements could be made to better address the problem?
12. What would be needed to make these improvements? By whom?
13. What types of serious health problems do PHCU staff or HEWs see during the month after a woman gives birth?
14. How is the problem addressed? By whom?
15. What improvements could be made to better address the problem?
16. What would be needed to make these improvements? By whom?

Question set 5. Maternal waiting areas

1. What do you see as reasons for women to use MWAs?
2. What are the reasons for women to not use MWAs?
3. What could be done to encourage more women to use MWAs?
4. What qualities of MWAs do you think are the most important in encouraging women to use them?
5. How can qualities of MWAs be sustained or improved?

*Prompt: role of government, PHCU, communities*

Before we close, do you have anything else you’d like to say about how you and the PHCU (including HEWs) work to promote the health of women and their babies?
